# Diversity Outbred Mice Identify Population-Based Exposure Thresholds and Genetic Factors that Influence Benzene-Induced Genotoxicity

**DOI:** 10.1289/ehp.1408202

**Published:** 2014-11-06

**Authors:** John E. French, Daniel M. Gatti, Daniel L. Morgan, Grace E. Kissling, Keith R. Shockley, Gabriel A. Knudsen, Kim G. Shepard, Herman C. Price, Deborah King, Kristine L. Witt, Lars C. Pedersen, Steven C. Munger, Karen L. Svenson, Gary A. Churchill

**Affiliations:** 1 Division of the National Toxicology Program, National Institute of Environmental Health Sciences (NIEHS), National Institutes of Health (NIH), Department of Health and Human Resources (DHHS), Research Triangle Park, North Carolina, USA; 2 The Jackson Laboratory, Bar Harbor, Maine, USA; 3 Division of Intramural Research, NIEHS, NIH, DHHS, Research Triangle Park, North Carolina, USA; 4 National Cancer Institute at NIEHS, NIH, DHHS, Research Triangle Park, North Carolina, USA; 5 Integrated Laboratory Systems Inc., Research Triangle Park, North Carolina, USA; 6 Alion Science and Technology, Research Triangle Park, North Carolina, USA

## Abstract

**Background:**

Inhalation of benzene at levels below the current exposure limit values leads to hematotoxicity in occupationally exposed workers.

**Objective:**

We sought to evaluate Diversity Outbred (DO) mice as a tool for exposure threshold assessment and to identify genetic factors that influence benzene-induced genotoxicity.

**Methods:**

We exposed male DO mice to benzene (0, 1, 10, or 100 ppm; 75 mice/exposure group) via inhalation for 28 days (6 hr/day for 5 days/week). The study was repeated using two independent cohorts of 300 animals each. We measured micronuclei frequency in reticulocytes from peripheral blood and bone marrow and applied benchmark concentration modeling to estimate exposure thresholds. We genotyped the mice and performed linkage analysis.

**Results:**

We observed a dose-dependent increase in benzene-induced chromosomal damage and estimated a benchmark concentration limit of 0.205 ppm benzene using DO mice. This estimate is an order of magnitude below the value estimated using B6C3F1 mice. We identified a locus on Chr 10 (31.87 Mb) that contained a pair of overexpressed sulfotransferases that were inversely correlated with genotoxicity.

**Conclusions:**

The genetically diverse DO mice provided a reproducible response to benzene exposure. The DO mice display interindividual variation in toxicity response and, as such, may more accurately reflect the range of response that is observed in human populations. Studies using DO mice can localize genetic associations with high precision. The identification of sulfotransferases as candidate genes suggests that DO mice may provide additional insight into benzene-induced genotoxicity.

**Citation:**

French JE, Gatti DM, Morgan DL, Kissling GE, Shockley KR, Knudsen GA, Shepard KG, Price HC, King D, Witt KL, Pedersen LC, Munger SC, Svenson KL, Churchill GA. 2015. Diversity Outbred mice identify population-based exposure thresholds and genetic factors that influence benzene-induced genotoxicity. Environ Health Perspect 123:237–245; http://dx.doi.org/10.1289/ehp.1408202

## Introduction

Benzene is a ubiquitous airborne pollutant that is classified as a carcinogen [International Agency for Research on Cancer 2012; National Toxicology Program (NTP) 2011a; U.S. Environmental Protection Agency (EPA) 1998]. Human exposure to benzene occurs mainly through inhalation in both environmental and occupational settings (Weisel 2010). The U.S. Occupational Safety and Health Administration has set an 8-hr, time-weighted average exposure limit of 1 ppm. However, workers exposed to benzene at air concentrations < 1 ppm still show evidence of hematotoxicity (Lan et al. 2004; McHale et al. 2012). Occupational exposures as low as 0.3 ppm have been shown to increase the risk of leukemia and myelodysplastic syndrome (Glass et al. 2003; Schnatter et al. 2012). At the molecular level, benzene exposure alters gene expression in peripheral blood cells (McHale et al. 2009), induces aneuploidy in hematopoietic progenitor cells (Zhang et al. 2012), and induces chromosome damage in myeloid progenitor cells (McHale et al. 2008). These and a multitude of other effects including AhR dysregulation, reduced immunosurveillance, and oxidative stress probably lead to benzene-induced hematologic malignancies (McHale et al. 2012).

Benzene metabolism and clearance varies significantly in the human population (Dorne et al. 2005; Hattis et al. 1987), and these differences may be influenced by both past or current co-exposures as well as genetic factors. Occupational studies of benzene exposure focusing on candidate genes have quantified the effects of polymorphisms on hematotoxicity and genotoxicity end points (Garte et al. 2008; Kim et al. 2007; Lan et al. 2005, 2009; Shen et al. 2006). However, the scope and impact of human genetic variation on toxicity responses remains poorly understood.

Exposure thresholds for suspected toxicants are typically determined using animal models. One of the most common mouse models used in toxicology assessments at the NTP is the B6C3F1 mouse (King-Herbert and Thayer 2006). These mice are derived by crossing two inbred strains—C57BL/6J and C3H/HeJ—and, like identical twins, all B6C3F1 mice are genetically identical (i.e., isogenic). The use of isogenic mice has been considered an advantage in toxicology studies because it should reduce the within-group variance, which in turn reduces the number of animals required to detect a specified level of response. However, isogenic mice fail to model the influence of genetic diversity on toxicity responses, a critical factor in human responses to toxicants. Furthermore, inadvertent selection of a strain with an idiosyncratic response could result in significant bias and compromise the reliability of safe exposure estimates.

There has been increased interest in incorporating genetic variability into human health risk assessments (King-Herbert et al. 2010; Zeise et al. 2013). One approach involves testing toxicants in mice that are genetically diverse rather than isogenic. Genetic diversity can be incorporated by using panels of inbred mice, such as the Mouse Diversity Panel (McClurg et al. 2007) and the Collaborative Cross (Threadgill et al. 2011), or by using populations of outbred mice (Yalcin et al. 2010). Outbred mice offer several advantages over inbred mice; like humans, they are genetically heterozygous and carry a complex mixture of alleles. Each animal in an outbred population is genetically unique, which leads to more efficient study designs compared with strain panels (Belknap 1998). However, many commercially available outbred stocks suffer from limited genetic diversity (Yalcin et al. 2010) and cryptic population structure (Aldinger et al. 2009).

The Diversity Outbred (DO) mice are a recently developed population derived from eight inbred founder strains (Svenson et al. 2012). The founder strains have been fully sequenced and contribute approximately 38 million single nucleotide polymorphisms (SNPs) and 7 million structural variants to the DO (Keane et al. 2011; Yalcin et al. 2011)—a level of genetic diversity that is similar to humans (Ideraabdullah et al. 2004). In the present study we used DO mice to assess a toxicity response to benzene exposure and to estimate a benchmark concentration (BMC) of benzene exposure that is likely to result in a 10% increase in chromosomal damage over baseline. We observed that, despite significant variation in benzene-induced chromosomal damage among DO mice, BMC estimates were repeatable across different study cohorts. Furthermore, estimates were lower by an order of magnitude compared with estimates derived from previous reports based on an isogenic mouse model. In addition to toxicity screening, the unique genetic structure of the DO mice can be used to perform high-resolution linkage mapping to identify genetic variants that determine individual responses to toxicant exposures. This study demonstrates the utility of DO mice for the estimation of exposure thresholds and for discovery of the genetic basis of variation in toxicant responses.

## Materials and Methods

*Benzene inhalation exposure*. We obtained 600 male DO mice (J:DO; stock no. 009376), between 3 and 5 weeks of age, from The Jackson Laboratory (Bar Harbor, ME). The mice were received in two cohorts of 300 mice each from the 7th and 8th generation of outbreeding, respectively, which were delivered 4 months apart. We provided the mice with NIH-31 diet (http://www.ors.od.nih.gov/sr/dvr/drs/nutrition/Documents/SpecsDiets/31.pdf) *ad libitum* and filtered tap water. Mice were singly housed in cages with irradiated, heat-treated hardwood bedding (Teklad, Indianapolis, IN). Mice were acclimated for 11–18 days and were 5–8 weeks of age at the start of the exposures. We randomized the mice to each exposure group after weight stratification such that the mean body weight was similar (± 20%) in each exposure group. We exposed 75 mice in each cohort to one of four benzene concentrations; 0 (filtered air), 1, 10, or 100 ppm by volume. We exposed the mice in whole-body inhalation chambers for 6 hr/day, 5 days/week for a total of 26–28 exposures. The mean daily exposure concentrations for cohort 1 were 1.001 ± 0.003, 10.07 ± 0.02, and 100.13 ± 0.11 ppm. For cohort 2, they were 1.000 ± 0.003, 9.99 ± 0.12, and 100.18 ± 0.10 ppm. Quality control data for the exposure chambers is available upon request from the corresponding author. We rotated cages within a rack and chamber over time using standard protocols (NTP 2011b; Office of Laboratory Animal Welfare 2002). We euthanized the mice by CO_2_ asphyxiation (95% CO_2_ + 5% O_2_) on the morning following the last exposure. These studies were conducted under NIH guidelines (Office of Laboratory Animal Welfare 2002) and were approved by the NIEHS Institutional Animal Care and Use Committee. Animal care and experimental design followed all applicable NIH protocols and standard operating procedures to alleviate animal suffering or deprivation.

*Tissue collection*. We collected blood before the first exposure via the tail vein and stored it in a heparin-containing Vacutainer® (Becton Dickinson and Co., Franklin Lakes, NJ). At euthanasia, we collected blood via cardiac puncture into EDTA Vacutainer® tubes. We collected bone marrow by aspirating the right femur with heat-inactivated fetal bovine serum for flow cytometry micronucleus analysis (Witt et al. 2008).

*Micronucleus assay*. We used micronucleus (MN) frequency in reticulocytes (RET) and mature erythrocytes (ERC) as a quantitative measure of chromosomal damage. Micronuclei are chromosomes or chromosomal fragments that fail to incorporate into either of the two daughter nuclei after karyokinesis. During formation of the reticulocyte in the bone marrow, micronuclei not extruded with the main nucleus are readily visualized and quantified, providing a simple, accurate assessment of chromosomal damage (Heddle 1973; Witt et al. 2008). We isolated RETs and ERCs from peripheral blood (PB) before the first exposure and in PB and bone marrow (BM) at euthanasia. Samples were processed immediately upon collection as described for the MicroFlow® BASIC kits (for mouse) (Litron Laboratories, Rochester, NY) and as described by Witt et al. (2008). We counted cells and assessed MN frequency in RETs and ERCs using flow cytometry. RETs were identified by the presence of an active transferrin receptor (CD71+) on the cell surface; CD71-negative cells were identified as mature ERCs. We evaluated approximately 20,000 RETs and 10^6^ mature ERCs for each animal (Kissling et al. 2007). In addition, the percentage of RETs (%RET) was determined in approximately 10^6^ ERCs. MN data were normalized to counts/1,000 cells. We calculated the means and SDs on the log scale and transformed them back to the original scale because MN-RETs and MN-ERCs are log-normally distributed. Here, we report the geometric mean of MN-RETs and MN-ERCs along with the antilog of the confidence limits (Bland and Altman 1996). We successfully measured MN-RET in PB for 592 mice and in BM for 570 mice.

*Statistical comparisons*. We estimated the effect of cohort and exposure concentration using two-way analysis of variance (ANOVA) on the log_2_-transformed values. We calculated the mean ± SD of MN-RET on a log scale and took the antilog. We report the geometric mean and confidence interval (CI) as the geometric mean ± 2 SD. We used Tukey’s honest significant difference to estimate phenotype differences between exposure groups (Hsu 1996).

*BMC modeling*. We used the MN-RET measurements from both cohorts, separately and combined, to estimate exposure thresholds using BMC modeling (Crump 1984). This method is recommended by the U.S. EPA’s Guidelines for Carcinogen Risk Assessment (U.S. EPA 2005) and is implemented in the U.S. EPA Benchmark Dose Software (BMDS, v2.4; U.S. EPA 2012b), which was used for all BMC analyses. We followed the methodology described in the Technical Guidance Document 2012 (U.S. EPA 2012a) for BMC modeling.

The benchmark response (BMR) is the minimum increase in MN-RETs above the control group mean that is considered to be biologically significant. BMR is used to define an associated BMC, which is the concentration of benzene that is expected to result in the specified BMR. We evaluated the BMC using a BMR defined as a 10% increase (BMR_10_) and 1-SD (BMR_1SD_) increase in MN-RET above the 0-ppm group mean. The benchmark concentration limit (BMCL) is the lower bound of a one-sided 95% CI around the BMC.

We fit the U.S. EPA recommended models for continuous data, including Hill, linear, second-order polynomial, power, and exponential models. The exponential model (Model 4 in BMDS) provided the best fit to the data (goodness of fit, *p* > 0.1) and was used to calculate the BMC and BMCL. The exponential continuous data model was

*μ*(*X*) *= a*[*c –* (*c* – *1*)*e^–bX^*], [1]

where *μ*(*X*) is median response at exposure concentration *X*, *a* is background response, *b* is slope, and *c* is an asymptote parameter (U.S. EPA 2012a). We used BMDS to fit the model using all exposure groups but found that the model fit was improved by removing the 100-ppm exposure group. As suggested by the U.S. EPA guidance, we fit the model using only the 0-, 1-, and 10-ppm groups. This choice was motivated by the desire to accurately model responses at the lower end of the exposure range.

We obtained exposure–response data for reticulocytes in bone marrow of B6C3F1 mice at 4 weeks from Farris et al. (1996). B6C3F1 used for this study were F_1_ mice derived by crossing female C57BL/6J and male C3H/HeJ mice. We calculated the BMCL for the B6C3F1 mice using the same methods as for DO mice.

Summary files for these analyses are available online (http://cgd.jax.org/datasets/phenotype/French_EHP_2014.shtml) and are listed in the Supplemental Material.

*Genotyping*. We collected a tail biopsy from each mouse and flash froze it in liquid nitrogen. We sent the tail tips to GeneSeek (Lincoln, NE) for DNA isolation and genotyping on the Mouse Universal Genotyping Array (MUGA) (Collaborative Cross Consortium 2012), which contains 7,854 probes distributed across the mouse genome. The mean (± SD) sample call rate was 94.7 ± 1.2%. Seven samples with allele calls rates < 90% were removed from the genotyping data. The genotype data are available in the Supplemental Files available online (http://cgd.jax.org/datasets/phenotype/French_EHP_2014.shtml).

*Linkage mapping*. Using the genotype data from the MUGA, we reconstructed the genomes of each DO mouse in terms of the founder haplotypes using a hidden Markov model (HMM) (Gatti et al. 2014). Briefly, at each marker, the HMM estimates the haplotype contribution of each of the eight founders to each mouse. We performed linkage mapping by regressing log(MN-RET/1,000 RETs) on the haplotype contributions at each marker. The regression model includes an adjustment for relatedness among individual DO mice (Cheng et al. 2011). We mapped log(MN-RET) in PB and BM using 144 animals with complete data from the two 100-ppm exposure groups. Linkage analysis provides a likelihood ratio statistic (LOD) at each marker and eight regression coefficients that estimate the additive effects of founder alleles. Genome-wide significance thresholds for linkage mapping were determined by analyzing 1,000 permutations of the phenotype data (Churchill and Doerge 1994). We used the Bayesian credible interval method to define the quantitative trait locus (QTL) peak support interval (Sen and Churchill 2001).

*Association mapping*. The eight DO founders have been fully sequenced (Keane et al. 2011; Yalcin et al. 2011). We used the haplotype reconstructions of the DO mice to impute the founder sequences onto the DO genomes within the Chr 10 linkage peak interval and mapped log(MN-RET) by additive regression on SNP genotypes (Gatti et al. 2014; Rat Genome Sequencing and Mapping Consortium et al. 2013).

*Heritability*. We estimated heritability based on the genetic and environmental variance components for preexposure peripheral blood MN-RET. Using the R package *regress* (Clifford and McCullagh, Commonwealth Scientific and Industrial Research Organization, Australia; R Foundation for Statistical Computing, Vienna, Austria), we fit a mixed model in which the covariance structure was a linear combination of the kinship matrix and the identity matrix and estimated the narrow-sense heritability as the ratio of additive genetic variance over total variance.

*Test for dominance*. We compared three models in order to test for dominance of the CAST/EiJ (CAST) allele at the Chr 10 QTL. We extracted the CAST allele proportion from the haplotype reconstructions at 31.8682 Mb on Chr 10. We coded the additive effect of the CAST allele as 0, 0.5, or 1, depending on whether the CAST allele was missing, heterozygous, or homozygous, respectively, in each mouse. We coded the dominant effect of the CAST allele as 0 if the mouse had no CAST allele and 1 if the mouse had at least one copy of the CAST allele. We fit an additive model, a dominant model, and a general model with both an additive and dominant effect. We performed a likelihood ratio test comparing the general model with the additive and dominant models separately.

*Founder gene expression*. We obtained founder gene expression in liver, kidney, and spleen for the genes in the Chr 10 QTL interval from the Gene Expression Survey of 26 Inbred Mouse Strains including the eight founder lines (Jackson Laboratory 2014). Briefly, expression was measured in the liver, kidney, and spleen of male and female (*n* = 5/sex) inbred mice from 26 inbred strains on the Illumina Sentrix Mouse 6 v1.1 platform (Illumina Inc., San Diego, CA). Probes containing Sanger SNPs (described below) were removed from the analysis. Reported values are log_2_-normalized intensity.

*Mouse genes and SNPs*. We obtained mouse genes from the Mouse Genome Database, v5.18 (Blake et al. 2014). We obtained the founder SNPs and structural variants from the Sanger Mouse Genomes Project, REL-1303 (Keane et al. 2011; Yalcin et al. 2011). All genomes coordinates are on Genome Reference Consortium Mouse Genome Build 38 (GRCm38; http://www.ncbi.nlm.nih.gov/projects/genome/assembly/grc/mouse/).

Supplemental Material and Files. All data from this study as has been posted at http://cgd.jax.org/datasets/phenotype/French_EHP_2014.shtml.

## Results

We exposed two independent cohorts of 300 male DO mice to benzene at 0, 1, 10, or 100 ppm and measured micronucleated reticulocytes and erythrocytes, markers of chromosomal damage in red blood cells. We estimated exposure thresholds using BMC modeling for each cohort separately and for the combined data. We performed linkage mapping to discover genomic loci that are associated with benzene-induced chromosomal damage in reticulocytes using DO mice in the combined 100-ppm exposure groups.

**Figure 1:**
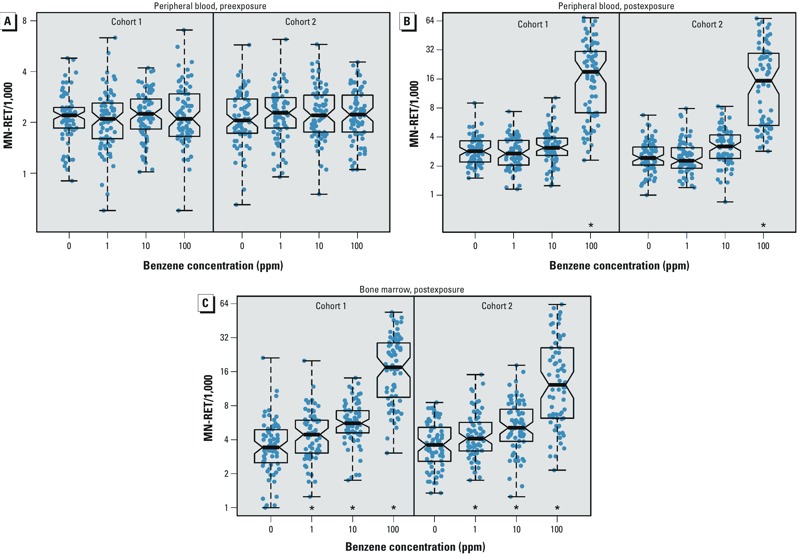
MN‑RET measurements were consistent between cohorts. (*A*) Preexposure blood MN‑RETs. Boxes represent the median and interquartile range, and whiskers cover the entire data range. (*B*) Postexposure blood MN‑RETs increased in the 100-ppm group. (*C*) Postexposure bone marrow MN‑RETs increased in a dose-dependent manner. *Approximately 1-unit increase in MN-RET/1,000 with each order of increase in benzene concentration.

*Benzene-induced chromosomal damage*. Before exposure, we measured MN-RET and MN-ERC in peripheral blood and found no difference between exposure groups (two-way ANOVA *p* = 0.56) or cohort (two-way ANOVA *p* = 0.44) (Figure 1A; see also Supplemental Material, Table S1). Data are presented only for peripheral blood MN-RET because the data are similar to blood MN-ERC values and the same quantitative trait locus is identified from both data sets (data not shown). The mean preexposure MN-RET was 2.17 (1.05, 4.47) MN-RET per 1,000 RETs (MN-RET/1,000). The estimated heritability of preexposure MN-RETs in PB was 0.65, which indicates that the genetic variance is roughly two times larger than the environmental variance.

After mice were exposed to benzene for 4 weeks, we measured MN-RET in peripheral blood and found a significant difference between exposure groups (two-way ANOVA *p* < 10^–16^). No significant differences were observed within dose groups across the two cohorts (Figure 1B; see also Supplemental Material, Table S2). The mean in the 0-ppm group was 2.68 MN-RET/1,000 (1.33–5.39). There was no difference in MN-RET means between the 0-ppm and the 1- or 10-ppm exposure groups (0- and 1-ppm Tukey HSD *p*-value = 0.93; 0 and 10 ppm *p* = 0.074). The mean in the 100-ppm exposure group increased to 14.6 MN-RET/1,000 (2.29–92.7, *p* = 5.03 × 10^–10^), corresponding to a 573% increase from baseline.

The Pearson correlations between pre- and postexposure PB MN-RET were 0.60, 0.63, 0.54, and 0.38 for the 0-, 1-, 10-, and 100-ppm groups, respectively. These values are consistent with the heritability estimate of 0.65 and again indicate that the error variance is roughly half of the genetic variance. Heritability of postexposure PB MN-RET was estimated to be 0.21.

At the end of the exposure period, we measured MN-RET in bone marrow and observed an increase with increasing exposure concentration (linear model in which bone marrow MN-RET was regressed on exposure concentration, *p* < 10^–16^) (Figure 1C; see also Supplemental Material, Table S3). Again there was no significant difference between cohorts (see Supplemental Material, Table S3). For the 1- and 10-ppm exposure groups, MN-RET/1,000 increased by roughly 1 unit with each order of magnitude increase in benzene concentration. In the 100-ppm exposure group, MN-RET/1,000 increased by 9 units above the 10-ppm group. Heritability of postexposure BM MN-RET was 0.035.

*BMC modeling*. We used the BM MN-RET data to estimate benzene exposure thresholds using the BMC modeling approach (Crump 1984). This approach involves fitting a model to the exposure–response data, selecting a biologically relevant toxicity response (i.e., BMR), matching this response to an exposure concentration (i.e., BMC) and estimating the lower bound on the CI (i.e., BMCL) as described in “Methods and Methods.”

**Figure 2:**
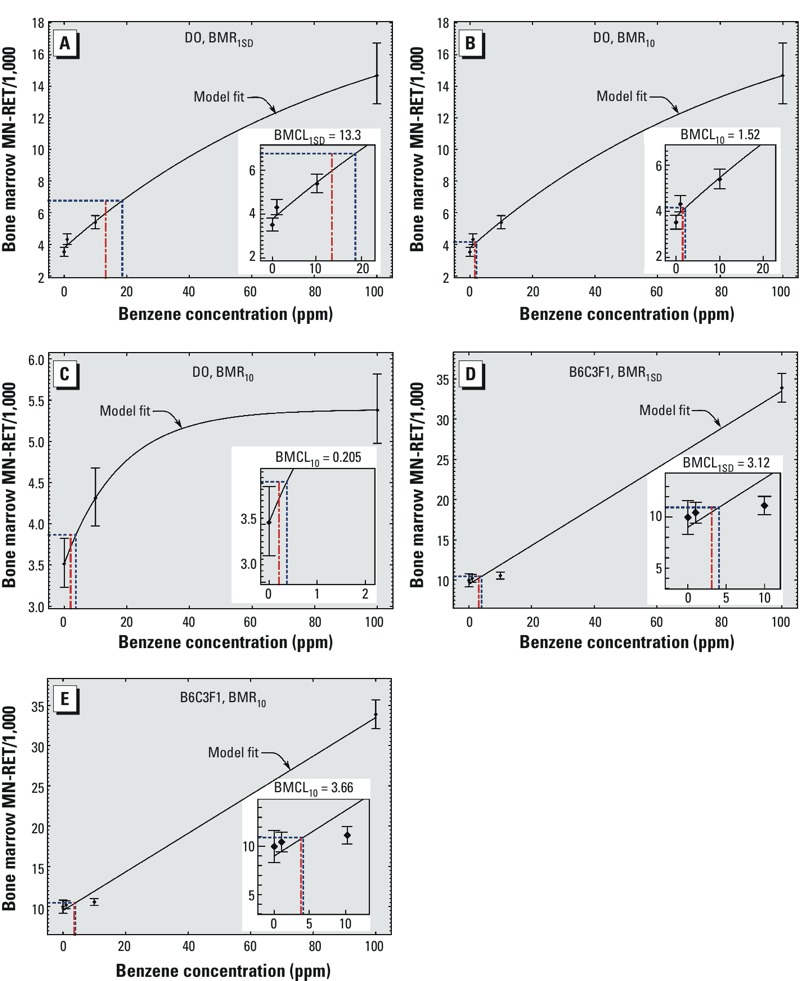
BMC modeling of bone marrow MN‑RET in DO mice using the 0-, 1-, 10-, and 100-ppm exposure groups to estimate a BMR_1SD_ (*A*) and a BMR_10_ (*B*). Values shown are the mean of each exposure group ± SE. Curved black lines represent the model fit, dashed blue lines represent the BMC, and red dot and dash lines indicate the BMCL. Insets show details of low concentration range. (*C*) BMC modeling of bone marrow MN‑RET in DO mice using the 0-, 1-, and 10-ppm exposure groups using a BMR_10_. BMC modeling of bone marrow PCE in B6C3F1 mice using a BMR_1SD_ (*D*) and a BMR_10_ (*E*).

We fit an exponential model to BM MN-RET using 570 mice in the combined data from all four exposure groups and estimated a BMCL_1SD_ of 13.3 (Table 1, Row 15, and Figure 2A) and a BMCL_10_ of 1.52 ppm (Table 1, Row 7, and Figure 2B). However, we observed a significant lack-of-fit statistic (*p*_lack-of-fit_ = 0.021). U.S. EPA guidelines indicate that it is acceptable to remove the highest concentration in order to improve the model fit at the lower concentrations where the BMCL will be determined (U.S. EPA 2012a). The reasoning behind this recommendation is that lack of fit may be indicative of different type of physiological response at higher exposures. We removed the 100-ppm group measurements and, using data from the 424 mice in the 0- through 10-ppm exposure groups, fit an exponential model to the data. We estimated a BMCL_10_ of 0.205 ppm (Table 1, Row 3, and Figure 2C). However, we were unable to calculate the BMCL_1SD_ in the 0- through 10-ppm groups because the mean MN-RET plus 1 SD of the 0-ppm group was greater than the 10-ppm group MN-RET mean. We repeated the benchmark dose analysis separately on each cohort of DO mice and obtained results consistent with the combined analysis (Table 1) to demonstrate reproducibility.

**Table 1:** BMCL for benzene-induced MN‑RET in DO and B6C3F1 mice.

Row	Strain/population	Doses (ppm)	AIC	BMR	BMCL
1	DO: Cohort 1	0–10	–81.2	10%	0.148
2	DO: Cohort 2	0–10	–88.3	10%	0.167
3	DO: All	0–10	–176	10%	0.205
4	B6C3F1	0–10	7.56	10%	NA
5	DO: Cohort 1	0–100	–47.2	10%	1.25
6	DO: Cohort 2	0–100	6.76	10%	1.46
7	DO: All	0–100	–37.2	10%	1.52
8	B6C3F1	0–100	47.8	10%	3.66
9	DO: Cohort 1	0–10	–81.2	1 SD	NA
10	DO: Cohort 2	0–10	–88.3	1 SD	NA
11	DO: All	0–10	–176	1 SD	NA
12	B6C3F1	0–10	7.56	1 SD	NA
13	DO: Cohort 1	0–100	–47.2	1 SD	9.87
14	DO: Cohort 2	0–100	6.76	1 SD	14.1
15	DO: All	0–100	–37.2	1 SD	13.3
16	B6C3F1	0–100	47.8	1 SD	3.12
Abbreviations: AIC, Akaike information criterion; NA, BMCL could not be calculated.					

We obtained bone marrow erythrocyte toxicity data from a previously published benzene inhalation study using the same study design in male B6C3F1 mice (Farris et al. 1996) and performed BMC modeling. Using the micronucleated polychromatic erythrocyte (MN-RET) data from bone marrow at 4 weeks, we estimated a BMCL_1SD_ of 3.12 (Table 1, Row 16, and Figure 2D) and a BMCL_10_ of 3.66 ppm (Table 1, Row 8, and Figure 2E). Both of these values were an order of magnitude higher than the BMCL_10_ estimated using DO mice. We were unable to estimate a BMCL_10_ using the 0-, 1-, and 10-ppm exposure groups because the control group mean MN-PCE plus 10% was greater than the 10-ppm group MN-RET mean.

*Linkage mapping analysis*. We genotyped each mouse at 7,854 markers and performed linkage mapping on postexposure MN-RET in blood and bone marrow using 144 animals with complete data in the combined 100-ppm exposure groups.

**Figure 3:**
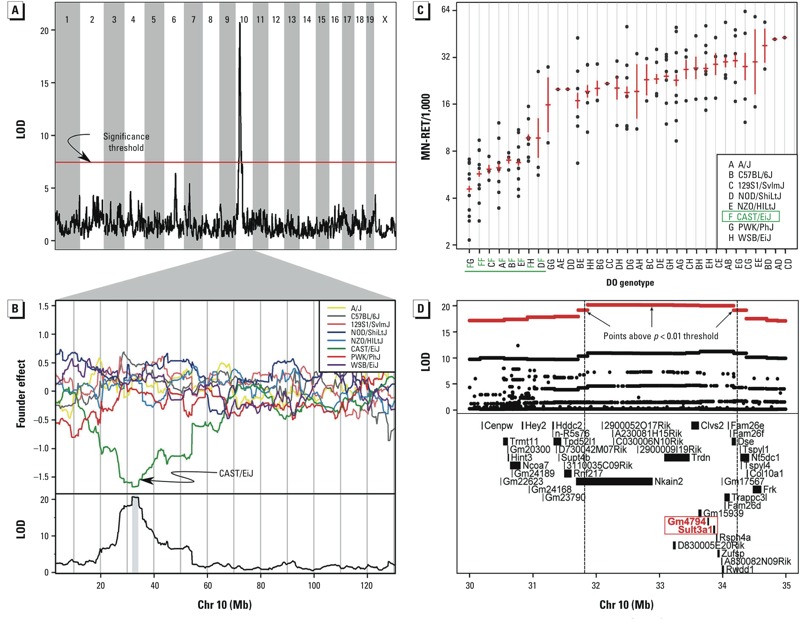
Linkage mapping of bone marrow MN‑RET in the 100-ppm exposure group revealed a significant QTL on Chr 10. (*A*) Plot of the LOD at each marker; the red line indicates the permutation-derived significance threshold of *p *= 0.05. (*B*, top) Plots of the effects of each of the eight DO founder alleles on Chr 10 (top) and the LOD score on Chr 10 (bottom); the CAST allele (green) is associated with lower MN‑RETs. (*C*) MN‑RET values by DO genotype at the marker with the maximum LOD score on Chr 10 (31.868 Mb). Data points indicate BM MN‑RET values for individual DO mice, and red lines show the mean ± SE of each genotype group. Genotypes are listed on the *x*-axis, with each DO founder represented by a letter; genotypes containing the CAST allele are shown in green. (*D*) Association mapping within the Chr 10 QTL interval. (*D*, top) Each data point shows the LOD score at one SNP; red data points indicate scores above the *p *< 0.01 threshold. (*D*, bottom) Genes in the QTL interval. Dashed vertical lines show the QTL support interval. *Sult3a1* and *GM4794* are highlighted in red to indicate their location.

We identified a single significant locus on Chr 10 shared by both PB and BM MN-RET (Figure 3A; see also Supplemental Material, Figure S1). We describe BM MN-RET findings here and provide results of PB analysis in Supplemental Material, Tables S1 and S2 and Figure S1. The maximum LOD score (20.7, *p* < 0.01) occurred at 31.87 Mb on Chr 10 (Figure 3B) and accounted for 48.7% of the phenotypic variance. We estimated the additive effect of each founder allele and observed that CAST/EiJ alleles are associated with lower levels of MN-RET (Figure 3B). Comparing the goodness-of-fit for genetic models of additive (*p* = 2.4 × 10^–7^) and dominant (*p* = 0.41) effects, we conclude that the CAST allele acts dominantly (Figure 3C). Mice carrying at least one copy of the CAST allele on Chr 10 are more resistant to benzene-induced chromosomal damage. We performed a second linkage mapping analysis with the Chr 10 QTL regressed out but did not find any additional QTL at the genome wide *p* < 0.05 level.

The support interval for the QTL on Chr 10 spans from 31.8 to 34.2 Mb and contains 21 annotated genes. To narrow this list of candidate genes, we carried out association mapping on imputed SNPs, looked for functional SNPs and small insertion–deletion polymorphisms in coding regions, examined external data resources to look for expression differences, and examined genomic sequence reads to look for large-scale structural polymorphisms. Several lines of evidence point to two of these genes, *Gm4794* (Gene model 4794) and *Sult3a1* (Sulfotransferase 3a1), as strong candidate genes for increased resistance to benzene-induced chromosomal damage.

We imputed the sequences of the individual DO genomes (Gatti et al. 2014) and performed association mapping at each SNP within the Chr 10 interval (Figure 3D). Although this approach can be helpful in narrowing the candidate gene list, in this case CAST contributes unique alleles that cover the entire QTL interval and these SNPs all have strong association with MN-RET. We found CAST-specific SNPs and small insertion–deletion polymorphisms in transcribed exons of 9 of the 21 candidate genes (Keane et al. 2011; Yalcin et al. 2011): *Clvs2, Dse, Gm15939, Gm4794, Rsph4a, Rwdd1, Sult3a1, Trdn,* and *Zufsp*.

**Figure 4:**
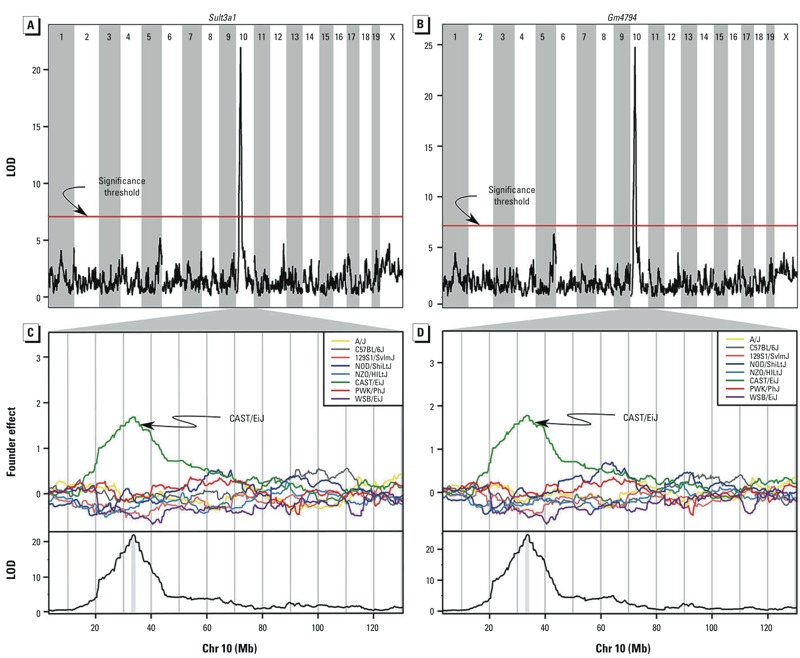
Linkage mapping of liver *Sult3a1* (*A,C*) and *Gm4794* (*B,D*) expression revealed a QTL on Chr 10 in the same location as the MN‑RET QTL. (*A*,*B*) Plot of the LOD at each marker; the red line indicates the permutation-derived significance threshold of *p *= 0.05. (*C*,*D*) Plots of the effects of each of the eight DO founder alleles on Chr 10 (top) and plots of the LOD score on Chr 10 (bottom). The CAST allele (green) is associated with higher liver *Sult3a1* and *Gm4794* expression.

We looked for CAST-specific expression differences in the DO founder strains and in an independent population of untreated DO mice (Munger et al. 2014). Three genes had differential expression in CAST; *Hddc2*, *Gm4794*, and *Sult3a1*. *Hddc2* (HD domain containing 2) had higher expression in liver, kidney, and spleen. However, in the liver expression QTL (eQTL) data, it had a local eQTL in which both the CAST and PWK alleles were associated with higher expression. This is not consistent with the MN-RET allele effects (Figure 3B). *Gm4794* and *Sult3a1* showed higher constitutive liver expression of CAST mice among the founder strains (see Supplemental Material, Figure S2) but were not expressed in kidney or spleen (data not shown). *Gm4794* and *Sult3a1* also had a strong liver eQTL for which the CAST allele increased expression (Figure 4). These eQTL are local and *cis*-acting.

We examined the genomic sequencing data of the founder strains in the QTL interval and found that CAST mice have a 350-Kb segmental duplication (33.52–33.87 Mb) that contains four genes: *Clvs2, Gm15939, Gm4794,* and *Sult3a1* (see Supplemental Material, Figure S3). *Clvs2* is expressed in neurons and does not appear to be expressed in liver or bone marrow (Wu et al. 2009). *Gm15939* is a predicted gene that is thought to be not transcribed. *Gm4794* is a paralog of *Sult3a1* that is transcribed and produces a protein product (ENSMUSP00000127159.1). It contains a sulfotransferase domain and shares 84% amino acid sequence identity with *Sult3a1*. There are no recombination events in our DO mice in the duplication region that could help to distinguish the effects of *Gm4794* from *Sult3a1*.

## Discussion

*Implications for BMC modeling*. The current U.S. EPA’s Integrated Risk Information System (IRIS) risk assessment benzene BMCL of 7.2 ppm is based on lymphocyte count data from 44 human subjects in an occupational exposure assessment (Rothman et al. 1996). In 2004, the same research consortium observed that workers exposed to < 1 ppm benzene had reduced white blood cell counts and circulating hematopoietic stem cells (Lan et al. 2004), indicating that additional research is necessary to determine appropriate risk thresholds for benzene exposure.

We performed BMC modeling on inhalation exposure data from DO mice and also on data from a previous study of B6C3F1 mice (Farris et al. 1996). Our estimated BMCL_10_ using DO mice, at 0.205 ppm benzene, was an order of magnitude lower than values obtained using B6C3F1 mice. The DO-based estimate of BMCL_10_ is consistent with observed exposure toxicity in human subjects (Lan et al. 2004) and thus may provide a more appropriate starting point for establishing exposure thresholds that protect the most sensitive human subpopulations.

There are several aspects in which our study may have improved inhalation exposure assessment for benzene. We examined exposure concentrations of 0, 1, 10, and 100 ppm. In retrospect it would have been preferable to add an exposure between 10 and 100 ppm to evaluate the fit of the standard dose–response model. In addition, because the estimated BMCL_10_ values fall below 1 ppm, an exposure group at a concentration between 0 and 1 ppm would increase our confidence in this estimate. However, low concentration exposures < 0.5 ppm are difficult to control in standard flow chambers. There may also be variation in respiration rates between DO mice and measuring the respiration rates may improve our ability to characterize the sources of variation in our study.

In the analysis of our data, we followed U.S. EPA–recommended guidelines for BMC modeling, but we encountered a number of problems. The significant lack of fit when the high-dose group was included suggested that the recommended models do not accurately reflect responses over the full range of exposures studied. Removing the high-exposure group data improved the overall model fit, but it would have been preferable to evaluate all of the data. In addition, when applying the recommended method for computing BMC, both our data and the B6C3F1 data failed to meet expectations implicit in the procedure. Extrapolation of risk estimates to low exposures is a challenging problem, and well-defined procedures are required to assure that consistent regulatory recommendations are provided. However, our experience in applying these procedures suggests that more research may be needed to identify new methods that conform to the realized properties of exposure–response data.

*Advantages of DO mice in toxicology*. The use of isogenic mice, such as B6C3F1, has been common practice for > 30 years (King-Herbert and Thayer 2006). Isogenic lines are assumed to produce lower variance and thus require smaller sample sizes compared with genetically diverse mice. This appears to be an advantage. However, isogenic mice do not reflect the variation in toxicity responses that we expect in a genetically diverse population such as humans. Furthermore, inadvertent selection of a strain (or F_1_ hybrid) model with an idiosyncratic response could result in significant bias and compromise the reliability of BMC estimates. These results may challenge current paradigms in toxicology testing, but these data suggest implicit benefits in modeling genetically diverse populations of test organisms.

The observed variation in MN-RETs in our study of DO mice was similar to isogenic B6C3F1 mice in the no-exposure group and ranged up to nine times greater (three times on SD scale) in the 100-ppm exposure conditions (see Supplemental Material, Table S3). There are difficulties in directly comparing studies that were carried out at different times and locations. However, we can obtain direct estimates of the genetic contribution to variance from the DO study. Heritability estimates suggest that sample sizes of DO mice required to obtain the same precision as with isogenic mice would need to be increased by a factor of three. The increased cost is offset by the potential to obtain more realistic and generalizable estimates of toxicity response in the genetically diverse populations. While it seems premature to make a general recommendation regarding sample sizes for DO-based toxicology studies, 75 animals per group is adequate to obtain reproducible estimates of BMC and BMCL.

DO mice encompass much greater genetic diversity than the classical inbred strains and commercial outbred stocks. Randomized breeding and genetic monitoring are employed to ensure constant population allele frequencies over time. In addition, the Collaborative Cross strains provide a resource of reproducible inbred animals from the same genetic background as the DO (Collaborative Cross Consortium 2012). This genetic reference population could be useful for validation and mechanistic studies of results obtained in DO mice.

In the present study the presence of CAST alleles on Chr 10 resulted in a significantly reduced response to benzene exposure. If, hypothetically, a mouse strain related to CAST had dominated mouse research in the same way that the C57BL/6 strain has, our assessment of exposure risk might look quite different. This illustrates the arbitrary nature of using a single inbred strain for exposure modeling. No single animal model can provide a perfect surrogate for evaluating toxicity in humans. However, the genetically diverse DO model provides a population-based assessment with levels of interindividual variation that more accurately reflect human populations.

*Identification of QTL associated with susceptibility to benzene exposure*. Using 144 DO mice in the combined 100-ppm exposure groups, we identified a region on Chr 10 in which mice carrying at least one CAST allele showed decreased chromosomal damage after exposure. Two genes, *Sult3a1* and *Gm4794,* showed increased hepatic expression in CAST/EiJ compared with other founder strains. We hypothesize that a genomic duplication specific to the CAST strain leads to increased expression of *Sult3a1* and *Gm4794* in the liver. These genes could play a protective role by sulfating benzene metabolites, such as phenol, before they can be transported to the bone marrow. In humans, sulfate conjugation of benzene metabolites such as phenol is thought to occur in the liver (McHale et al. 2012). Although humans lack a direct ortholog of *Sult3a1*, the human sulfotransferase with the closest amino acid similarity is a phenol sulfotransferase called SULT1A1 (Brix et al. 1999; Gamage et al. 2006). Humans contain between one and five copies of *SULT1A1* (Gaedigk et al. 2012; Hebbring et al. 2007; Yu et al. 2013), and our results suggest that copy number variation could be associated with the variation in benzene-induced toxicity in humans.

We did not find any significant association near genes that are traditionally associated with benzene metabolism—such as *Cyp2e1*, *Ephx1*, *Sult1a1*, *Mpo*, and *Nqo1*—in the 100-ppm exposure group. This may be due to a lack of functional polymorphisms in these genes in the DO, to the relative importance of these genes to benzene metabolism and clearance in the 100-ppm exposure group, or to differences in benzene metabolism between mice and humans.

## Conclusions

We have demonstrated that DO mice experience a reproducible dose-dependent increase in bone marrow MN-RET after benzene exposure by inhalation. We carried out a standard BMC modeling analysis using the DO mice and found that BMCL_10_ estimates were lower by an order of magnitude compared with an isogenic mouse model. We used genetic mapping analysis of DO mice to identify a locus on Chr 10 that is associated with benzene-induced genotoxicity. Analysis of gene expression and genomic sequencing data provide evidence in support of sulfotransferases as candidate genes underlying the QTL effect.

## Supplemental Material

(2.2 MB) PDF
