# Supplementary material for: Diversity Outbred Mice Identify Population-Based Exposure Thresholds and Genetic Factors that Influence Benzene-Induced Genotoxicity
Source: Environ Health Perspect. 2014 Nov 6;123(3):237–45. doi: 10.1289/ehp.1408202 (PMC4348743; doi:10.1289/ehp.1408202)
Supplement: (2.2 MB) PDF [file ehp.1408202.s001.508.pdf]

Supplemental Material

Diversity Outbred Mice Identify Population-Based Exposure Thresholds and Genetic Factors that Influence Benzene-Induced Genotoxicity

John E. French, Daniel M. Gatti, Daniel L. Morgan, Grace E. Kissling, Keith R. Shockley, Gabriel A. Knudsen, Kim G. Shepard, Herman C. Price, Deborah King, Kristine L. Witt, Lars C. Pedersen, Steven C. Munger, Karen L. Svenson, and Gary A. Churchill

Additional Files

The Supplemental Tables and Figures presented below and the following Supplemental Material files and relevant information are also available at:
http://do.jax.org/publications/French_EHP_2014.shtml

File Listing

French_etal_BMDS_files.zip
Benchmark dose modeling software inputs and outputs. This file contains:
1. French_et_at_benzene.ssn: BMDS modeling session file.
2. B6C3F1_BMDS_output.xlsx: BMDS output for B6C3F1 mice.
3. DO_BMDS_output.xlsx: BMDS output for DO mice.
4. Benzene_0_100_both_cohorts.dax: MN-RET data for both cohorts; 0, 1, 10 & 100 ppm.
5. benzene_0_100_cohort1.dax: MN-RET data for cohort 1; 0, 1, 10 & 100 ppm.
6. benzene_0_100_cohort2.dax: MN-RET data for cohorts 2; 0, 1, 10 & 100 ppm.
7. Benzene_0_10_both_cohorts.dax: MN-RET data for both cohorts; 0, 1 & 10 ppm.
8. benzene_0_10_cohort1.dax: MN-RET data for cohort 1; 0, 1 & 10 ppm.
9. benzene_0_10_cohort2.dax: MN-RET data for cohort 2; 0, 1 & 10 ppm.
10. benzene_B6C3F1.dax: MN-RET data for B6C3F1; 0, 1, 10 & 100 ppm.
11. benzene_B6C3F1_0_10ppm.dax: MN-RET data for B6C3F1; 0, 1 & 10 ppm.
12. benzene_EXP_1SD_LogNorm.opt: Modeling options file for DO: BMR1SD.
13. benzene_EXP_10perc_LogNorm.opt: Modeling options file for DO: BMR10.
14. B6C3F1_EXP_BMR1SD.opt: Modeling options file for B6C3F1: BMR1SD.
15. B6C3F1_EXP_BMR10.opt: Modeling options file for B6C3F1: BMR10.

French_etal_MNRET_Data.xlsx:
Hematology phenotype measurements on the DO mice used in this study.

Alion Mouse 31jan2012_FinalReport.txt:
Allele calls and array intensities for the DO mice used in this study.

Sample_Map.txt:
DO mouse sample names in the genotype file.

French_etal_genoprobs.zip:
Haplotype probabilities for 593 DO mice.

French_etal_QTL_Results.zip:
QTL mapping results from R.
The first file: benzene.dose100.QTL.Rdata
QTL results for the phenotypes, including blood MN-RET and bone marrow MN-RET.
The second file: benzene.dose100.perms.QTL.Rdata
Permutations for the phenotypes.

Table S1. Pre-exposure peripheral blood MN-RET / 1000 RETs.

Benzene (ppm)	Cohort 1 N	Cohort 1 Mean (C.I.)	Cohort 2 N	Cohort 2 Mean (C.I.)	Cohort p-value*	All samples N	All samples Mean (C.I.)	Exposure group p-value**
0	73	2.14 (1.094, 4.17)	75	2.10 (1.02, 4.32)	0.734	148	2.12 (1.06, 4.24)	NA
1	74	2.05 (0.905, 4.65)	74	2.20 (1.09, 4.44)	0.272	148	2.12 (0.99, 4.55)	1.000
10	75	2.20 (1.153, 4.18)	75	2.26 (1.10, 4.64)	0.625	150	2.23 (1.13, 4.40)	0.625
100	75	2.18 (0.964, 4.91)	74	2.21 (1.09, 4.48)	0.798	149	2.19 (1.03, 4.69)	0.826

*Student's T-test between cohort 1 and 2.

**Tukey honest significant difference between exposure group and 0 ppm group.

C.I. indicates the confidence interval transformed from the log scale.

Table S2. Post-exposure peripheral blood MN-RET / 1000 RETs.

Benzene (ppm)	Cohort 1 N	Cohort 1 Mean (C.I.)	Cohort 2 N	Cohort 2 Mean (C.I.)	Cohort p-value*	All samples N	All samples Mean (C.I.)	Exposure group p-value**
0	74	2.87 (1.51, 5.45)	74	2.50 (1.20, 5.19)	0.016	148	2.68 (1.33, 5.39)	NA
1	75	2.71 (1.30, 5.65)	74	2.44 (1.11, 5.37)	0.089	149	2.57 (1.19, 5.54)	0.930
10	75	3.12 (1.43, 6.80)	73	3.15 (1.31, 7.59)	0.882	148	3.14 (1.37, 7.17)	0.074
100	74	15.82 (2.65, 94.34)	71	13.39 (1.98, 90.63)	0.279	145	14.58 (2.29, 92.72)	5.031E-10

*Student's T-test between cohort 1 and 2.

**Tukey honest significant difference between exposure group and 0 ppm group.

C.I. indicates the confidence interval transformed from the log scale.

Table S3. Post-exposure bone marrow MN-RET / 1000 RETs.

Benzene (ppm)	Cohort 1 N	Cohort 1 Mean (C.I.)	Cohort 2 N	Cohort 2 Mean (C.I.)	Cohort p-value*	All samples N	All samples Mean (C.I.)	Exposure group p-value**
0	70	3.46 (1.15, 10.4)	72	3.57 (1.40, 9.11)	0.699	142	3.51 (1.27, 9.72)	NA
1	65	4.33 (1.57, 11.9)	71	4.30 (1.71, 10.83)	0.934	136	4.31 (1.65, 11.30)	0.018
10	75	5.56 (2.38, 13.0)	71	5.20 (1.80, 15.01)	0.398	146	5.38 (2.07, 14.02)	6.426E-09
100	75	16.77 (4.26, 66.0)	70	12.73 (2.20, 73.52)	0.038	145	14.68 (3.01, 71.70)	4.763E-10

*Student's T-test between cohort 1 and 2.

**Tukey honest significant difference between exposure group and 0 ppm group.

C.I. indicates the confidence interval transformed from the log scale.

Figure S1

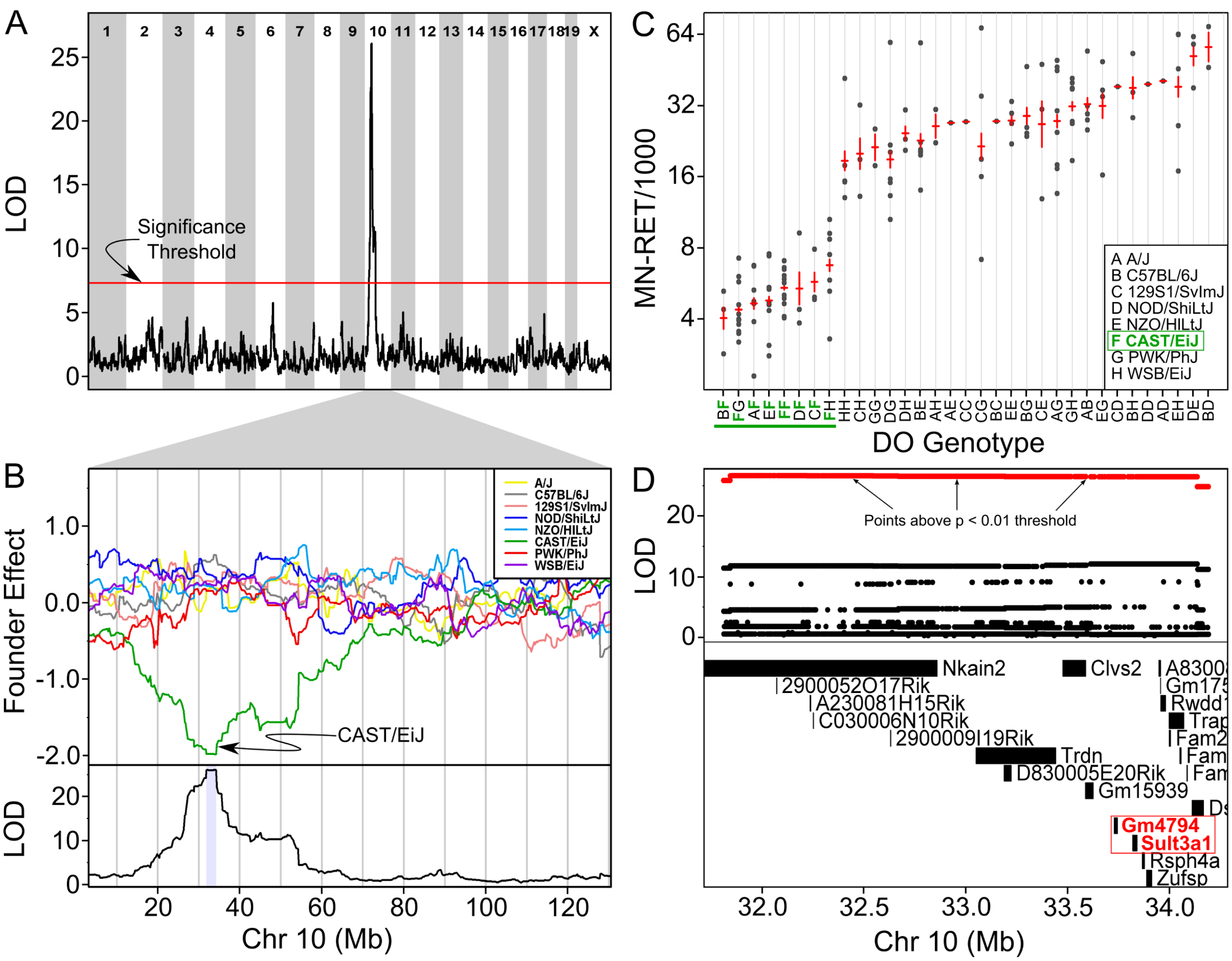

Figure S1. Linkage mapping of peripheral blood MN-RET in the 100 ppm exposure group reveals a significant QTL on Chr10. (A) Solid black line plots the LOD at each marker. Horizontal red line is permutation derived significance threshold of p = 0.05. (B) Upper panel plots the effects of each of the eight DO founder alleles on Chr 10. The CAST allele (green line) is associated with lower MN-RETs. Lower panel plots the LOD score on Chr 10. (C) BM MN-RET values versus DO genotype at the marker with the maximum LOD score on Chr 10 (33.648 Mb). Each dot shows the MN-RET value for a single DO mouse. Solid red lines are mean +/- standard error of each genotype. Genotypes are listed on the X-axis, with each DO founder represented by a letter. Genotypes containing the CAST allele are shown in green and boldface text and are underlined. DO mice with at least one copy of the CAST/EiJ allele on Chr 10, indicated by an “F” in the genotype, have lower MN-RET values. (D) Association mapping within the Chr 10 QTL interval. Each dot shows the LOD score at one SNP. Dots in red (or light grey when the image is viewed in greyscale) are above the p < 0.01 threshold. Lower panel shows the genes in the QTL interval. Dashed vertical lines show the QTL support interval. *Sult3a1* and *GM4794* are colored red and surrounded by a red box to highlight their location.

Figure S2

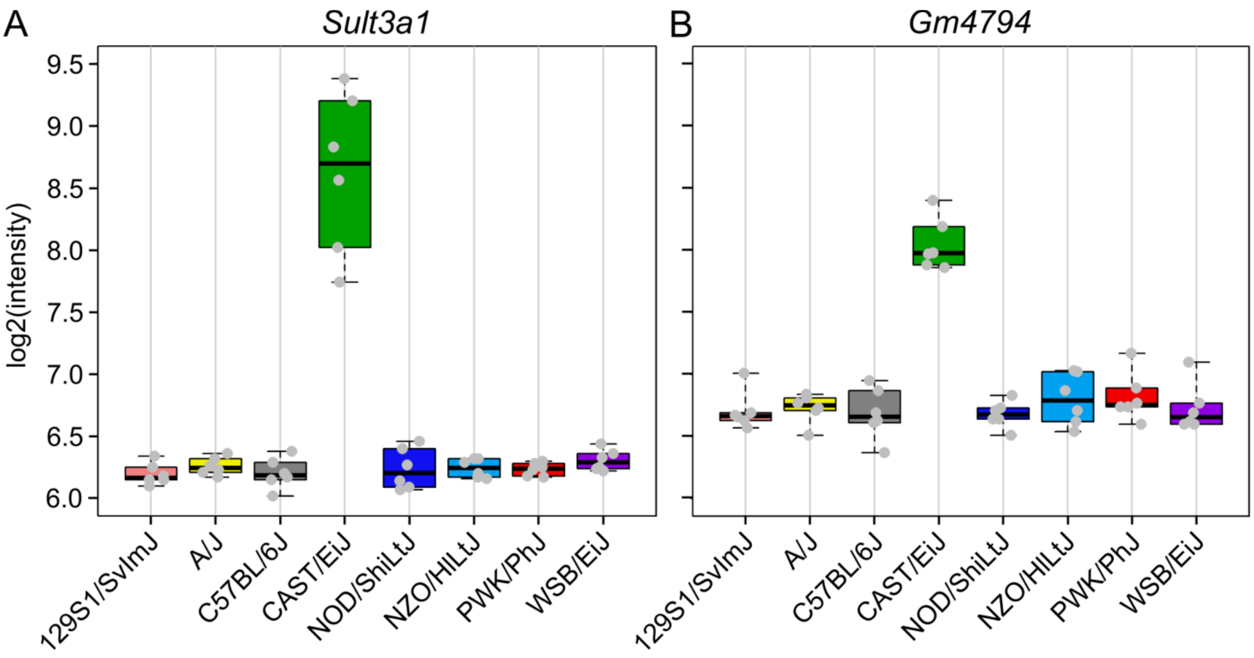

Figure S2. Constitutive liver S*ult3a1* (A) and *Gm4794* (B) expression in males from the eight DO founders is higher in CAST/EiJ mice. Boxes show the median and interquartile range of each strain. Whiskers show the range (i.e. the minimum and the maximum). Solid grey dots indicate the log_2 expression value of individual mice.

Figure S3

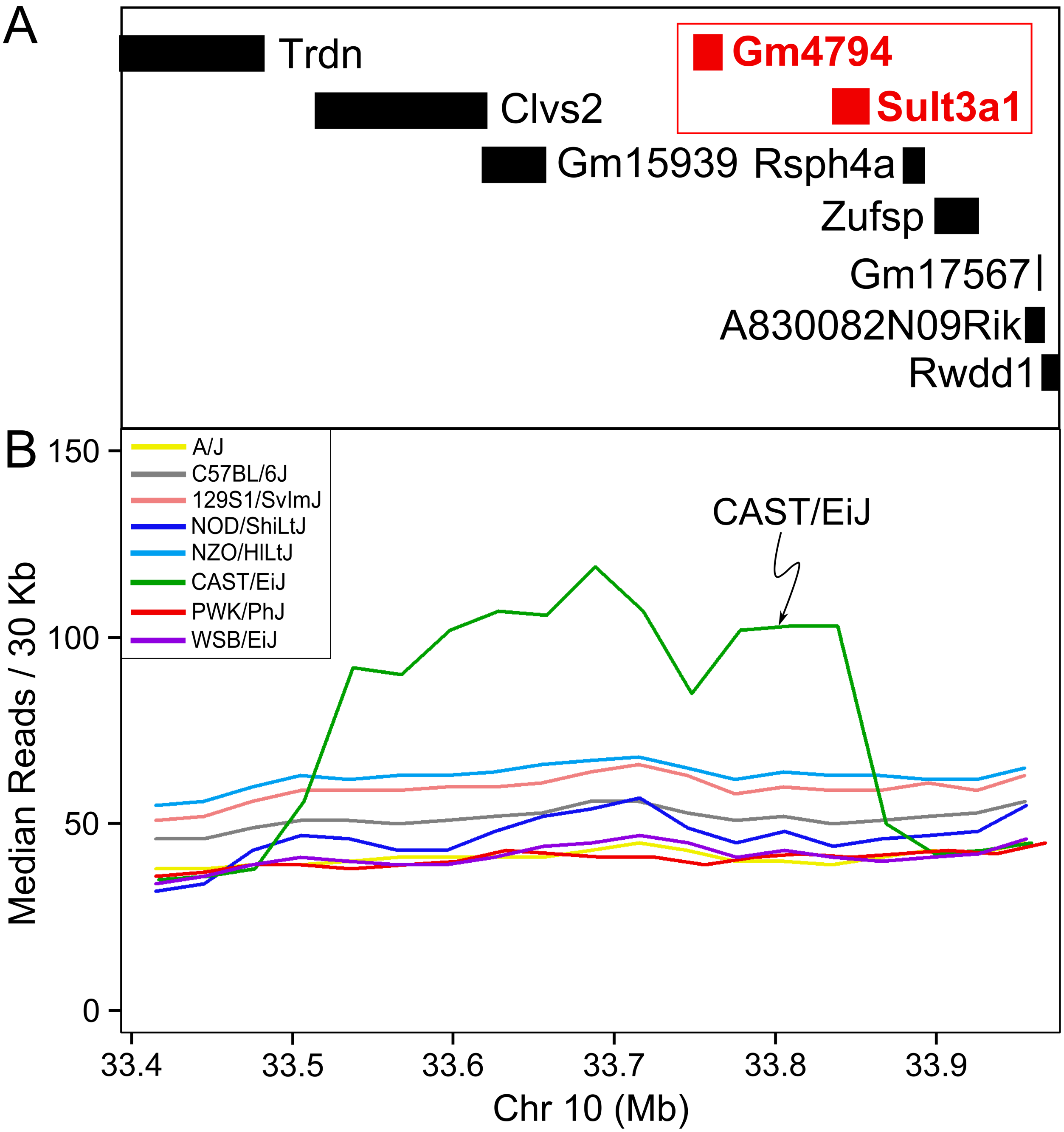

Figure S3. (A) Genes in the Chr 10 QTL interval near the CAST copy number gain. *Sult3a1* and *Gm4794* are highlighted in bold, red text and surrounded by a rectangle. (B) DNA-seq read depth for the eight DO founder in the region where CAST/EiJ (green) contains a copy number gain.
